# Alternative Methodology for Cortisol Evaluation Before and After Sheep Shearing Using Raman Spectroscopy: A Feasibility Study

**DOI:** 10.3390/ani15192776

**Published:** 2025-09-23

**Authors:** Giuseppe Acri, Barbara Testagrossa, Alberto Scoglio, Alessandro Attanzio, Francesca Arfuso, Maria Rizzo, Giuseppe Piccione, Claudia Giannetto

**Affiliations:** 1 Department of Biomedical, Dental, Morphological and Functional Imaging Sciences, University of Messina, 98125 Messina, Italy; gacri@unime.it (G.A.); alberto.scoglio@studenti.unime.it (A.S.); 2 Department of Biological, Chemical and Pharmaceutical Sciences and Technologies, University of Palermo, 90123 Palermo, Italy; alessandro.attanzio@unipa.it; 3 Department of Veterinary Science, University of Messina, 98168 Messina, Italy; farfuso@unime.it (F.A.); rizzom@unime.it (M.R.); gpiccione@unime.it (G.P.); clgiannetto@unime.it (C.G.)

**Keywords:** Raman spectroscopy, sheep shearing, cortisol, vibrational band analysis

## Abstract

This research proposed a feasibility study for a qualitative and semiquantitative analysis of sheep serum using Raman Spectroscopy analysis as an alternative to standard methodologies. Raman Spectroscopy was used to obtain information about molecular vibrations that can provide information about the behavior of specific variations in the protein network. This study was conducted during the traditional vernal shearing procedure in Sicilian sheep breeding. Twenty female sheep were randomly chosen from a one-hundred Comisana-bred flock habituated to the handling required for shearing and venipuncture. Animals had a mean body weight of 52.35 ± 4.55 kg, were aged between 2 and 3 years old, and were clinically healthy with no evidence of disease and free from internal and external parasites. All animals were shorn on the same day by hand using traditional shearing scissors in a 15 m × 10 m pen. The animals were released into an adjacent pen at the end of the shearing procedure. For each animal, blood samples were collected through jugular venipuncture into a vacutainer tube with a clot activator (Terumo Corporation, Japan) immediately before and 5 and 60 min after the end of the shearing procedure. On the obtained sera, ELISA and Raman spectroscopy analyses were performed to evaluate cortisol concentrations. The band area corresponding to the cortisol vibration mode was identified in the 1300–1366 cm^−1^ band. The Raman spectra obtained during the various protocol data points showed the same trend, with differences in the intensity of the band area 1300–1366 cm^−1^. A positive correlation was found between ELISA and Raman assessment in all experimental conditions. The obtained results demonstrate that Raman spectroscopy analysis could be a suitable tool for biomolecule identification. This study demonstrated that this technique provides useful information for understanding sheep responses to stress induced by management conditions.

## Simple Summary

Raman Spectroscopy is a rapid and alternative methodology applied in many biological tissues for the assessment of their compounds. The aim of the present study was to compare the serum cortisol levels assessed by means of an ELISA kit and Raman Spectroscopy. To do this, serum was collected in sheep subjected to shearing before and 5 and 60 min after the procedure; this is well known to be stressful for these animals. The obtained results demonstrate the feasibility of Raman spectroscopy analysis for the identification of cortisol in sheep serum. Further studies are necessary to verify its applicability to other physiological parameters in this species.

## 1. Introduction

Blood is the tissue most investigated to assess an animal’s health status. In particular, evaluating the animal’s hematological and hematochemical profiles is a useful and sensitive index to check physiological and pathological modifications [1]. The most commonly measured biomarkers for assessing physiological stress are the glucocorticoids, which indicate the activity of the hypothalamic–pituitary–adrenal (HPA) axis [2,3,4,5]. Glucocorticoids are at the crossroads of many biological pathways. They influence metabolism, increasing blood glucose concentrations and stimulating the use of amino acids from endogenous stores. The principal glucocorticoid is cortisol, which is secreted by the adrenals in response to acute and chronic stress, interpreted as a means to mobilize energy in the face of a threatening situation [6]. For this reason, it is often used as a stress indicator in animal behavior research. In sheep, cortisol has been measured in a variety of non-blood matrices, with varying results in terms of the relationship to concentrations in blood [7,8,9].

Detecting serum concentrations of various molecules relies on using specific immunoenzymatic kits (ELISA), radioimmunoassays (RIAs), or chromatographic techniques. These methods require a certain sample volume, several reagents, and long incubation and washing phases [10]. Conversely, Raman spectroscopy, in addition to be easy to perform, does not require reagents, reduces long-term costs, and provides extensive information on different biomarkers, including those measured to monitor stress variations, as demonstrated in previous studies where Raman spectroscopy has been proposed as a valid alternative tool for monitoring stress responses in human and animal biological samples, due to its ability to detect molecular changes associated with stress conditions [11,12,13,14,15]. It proves to be a method capable of enriching data from traditional ELISA measurements, offering a complementary and more versatile solution.

This optical technique is based on the irradiation of a sample with a laser beam, which produces a vibrational spectrum characteristic of the analyzed molecules. This spectrum contains highly specific information about the structure, the symmetry, and the chemical bonds of the molecules [16,17]. In recent years, this spectroscopic approach has been recognized as a key technology in the field of clinical diagnostics [18], demonstrating remarkable versatility in applications such as the diagnosis of tumors [19,20,21,22,23], inflammatory diseases [24,25], and neurodegenerative and infectious diseases [26,27,28,29]. Beyond the clinical context, Raman spectroscopy has also found wide application not only in medicine but also in multiple areas of basic and applied research, ranging from physics and chemistry to biology [30,31,32]. In addition to this wide-ranging relevance, Raman spectrum analysis allows the acquisition of detailed information about complex molecular systems, such as biological fluids. Among these, serum represents a prototypal example, being composed of a heterogeneous mix of electrolytes, proteins, carbohydrates, lipids, and metabolites [17,33,34]. Each of these biochemical substances exhibits unique spectroscopic fingerprints, and this feature enables Raman spectroscopy not only to discriminate between different molecular species but also to carry out the simultaneous analysis of multiple biomarkers and metabolites within just a few minutes, including cortisol.

In veterinary medicine, Raman spectroscopy has been applied for the identification of peripheral markers of fatigue during exercise and for quantifying serum total lipids and tryptophan concentrations during a standardized obstacle course in horses [35,36]. In dogs, a preliminary study for the application of Raman Spectroscopy for the identification of leishmania-infected dogs has been performed [37]. Although an ELISA represents the gold standard for cortisol quantification, we hypothesize that Raman spectroscopy may provide a rapid and non-destructive tool. If this technique correlates with the ELISA results, then it could represent a reliable alternative methodology for cortisol monitoring, which could be used in conjunction with the gold standard. Therefore, the aim of this study is to evaluate the use of Raman spectroscopy to monitor changes in serum cortisol levels recorded before and after sheep shearing, a stressful event known to increase this stress biomarker. The obtained results were compared with those found by using the ELISA methodology.

## 2. Materials and Methods

### 2.1. Experimental Animals

Twenty Comisana breed sheep aged between 2 and 3 years old, with a mean body weight of 52.35 ± 4.55 kg, belonging to a flock living 9 m above sea level, in Sicily, Italy (40°410 N; 14°260 E), were enrolled in the study. Comisana is a very popular dairy sheep breed, raised for its excellent, high-quality milk production. It originates from Sicily, takes its name from the town of Comiso, and is also widely distributed on farms in neighboring regions. Before the start of the study, the animals were subjected to a clinical exam to exclude diseases and the presence of internal and external parasites; only clinically healthy animals were included. All animals were regularly subjected to health checks for the prevention and prophylaxis of transmissible diseases, as required by Italian law. All flock animals were kept on natural pasture during the morning hours and were put in a pen during the night hours. The sheep had free access to water and hay (CP 11.1% and 7.2 MJ ME/kg DM), and during the recovery in the pen, 200 g/sheep of a commercial concentrate (crude protein (CP) 20.4% and 12.5 MJ metabolizable energy (ME)/kg dry matter (DM)) was added to their ration.

### 2.2. Shearing Procedure

All enrolled sheep were familiar with venipuncture and with the shearing procedure. In Sicily, in sheep breeding, this procedure is performed in late spring once a year. All animals were sheared on the same day, in June. The procedure started at 7:00 a.m. and took about 1 h and 50 min to complete the procedure on all sheep. During the experimental period, thermohygrometric recordings were performed. Ambient temperature and relative humidity were within the season’s local range (28 ± 2 °C and 64 ± 6%, respectively). Shearing was executed by hand using traditional shearing scissors in a 15 m × 10 m pen, and it lasted approximately 5 min for each sheep. At the end of the shearing procedure, the animals were released into an adjacent enclosure after being marked with a progressive number through an appropriate colored spray to identify them.

### 2.3. Blood Sampling

Blood samples were collected by jugular venipuncture using a vacutainer tube containing clot activator (Terumo Corporation, Tokyo, Japan) immediately before (PRE) and 5 min (POST) and 60 min (POST60) after the end of shearing. A total of 40 s was necessary to collect the samples from each sheep. A total of 10 min was necessary to collect blood samples from two different animals. All samples were left to clot at 4 °C overnight and were then centrifuged at 1000× *g* for 20 min at 2–8 °C. All the obtained sera were non-hemolyzed and then divided into two aliquots that were analyzed with two different techniques.

An ELISA kit specific for ovine species was used for the assessment of serum cortisol levels (Bovine/Sheep Cortisol ELISA kit, Elabscience Biotechnology Inc. Kampenhout, Belgium) using a microwell plate reader (Sirio, SEAC, Florence, Italy). Calibrators and samples were run in duplicate, and samples exhibited parallel displacement to the standard curve for both ELISA analyses. Both the intra- and the inter-assay coefficients of variation were 0.05.

### 2.4. Raman Spectroscopy Analysis

Immediately after the separation of the serum, an aliquot was promptly transported under refrigerated conditions to the Physics Laboratory of the Department of Biomedical, Dental, Morphological and Functional Imaging Sciences, University of Messina, for Raman analysis. All serum aliquots were stored at −20 °C and thawed only once, immediately prior to analysis. Raman measurements were performed using a diode laser with an excitation wavelength of 785 nm mounted on a DXR-SmartRaman spectrometer (Thermo Fisher Scientific, Waltham, MA, USA). Before measurements, the instrument was calibrated using standard wavelength samples provided by the manufacturer. Raman analysis was conducted on liquid serum samples. Vials containing 200 µL of serum per sheep were prepared and placed in the sample holder and analyzed using the 180-degree sampling accessory (Thermo Fisher Scientific, Waltham, MA, USA). Each Raman spectrum was acquired in the wavenumber range of 3300–400 cm^−1^ with a resolution of approximately 2 cm^−1^. The irradiation was performed using a laser power of 24 mW, coming out from a circular aperture 50 µm in diameter (pinhole). To improve the signal-to-noise ratio (S/N), each spectrum was obtained through a series of 16 exposures, each lasting 60.0 s, for a total acquisition time of 16 min per spectrum. All acquired Raman spectra were stored in SPA format, and post-processing analysis was performed using the DXR-SmartRaman spectrometer software (OMNIC for Dispersive Raman 9.1.24, Copyright © 1992–2012 Thermo Fisher Scientific Inc., Waltham, MA, USA).

In order to compensate for eventual technical and/or sample variations, a manual baseline correction was applied to each Raman spectrum. It is important to underline that noise and fluorescence can influence, in a negative manner, Raman spectra. Based on the literature, a filter methodology that uses a curve fitting technique improves S/N and reduces the interferences coming from noise sources. The optimal degree of the polynomial regression is 3 (cubic polynomial interpolation algorithm, spline) [38]. This process allowed the baseline to be subtracted from the original spectrum, obtaining a corrected Raman spectrum that is easier to interpret.

Subsequently, all Raman spectra were normalized using vector normalization [39]. In this procedure, each Raman intensity, associated with a specific Raman shift, was divided by a normalization factor (norm), calculated as the square root of the sum of the squared intensities of the spectrum:

(1) $$norm=\sqrt{\left(s_{1}^{2}+s_{2}^{2}+\ldots +s_{n}^{2}\right)}$$

From the normalized spectra, the spectral range of 1300–1366 cm^−1^ was considered for evaluation, corresponding to the C-C-C symmetric stretching in the cortisol A-ring, as reported in the literature [40,41]. Table 1 indicates the main vibrational bands and peaks and their tentative assignment, based on literature.

### 2.5. Statistical Analysis

The band centered at 1300–1366 cm^−1^ was perfectly visible in all the acquired spectra and did not require any deconvolution. This band was used to evaluate the changes in biochemical serum compounds depending on shearing.

One-way repeated measures analysis of variance (ANOVA) was applied to evaluate the modifications of band area and the serum concentrations of cortisol, due to shearing (*p* < 0.05 was considered statistically significant). Bonferroni’s multiple tests were applied for post-hoc comparison. A linear regression model (y = a + bx) was applied to determine the degree of correlation between the area of the band and the serum concentrations, obtained by using the ELISA method, of the investigated parameter.

Pearson’s correlation analysis was applied to assess the correlation between the data obtained with the ELISA test and Raman spectroscopy during the various data points of the experimental period. A linear regression model (y = a + bx) was applied to investigate the degree of correlation between the two procedures. *p*-values < 0.05 were considered statistically significant. Statistical analysis was performed by means of the software Prism v. 9.00 (Graphpad Software Ltd., San Diego, CA, USA, 2020).

## 3. Results

**Figure 1:**
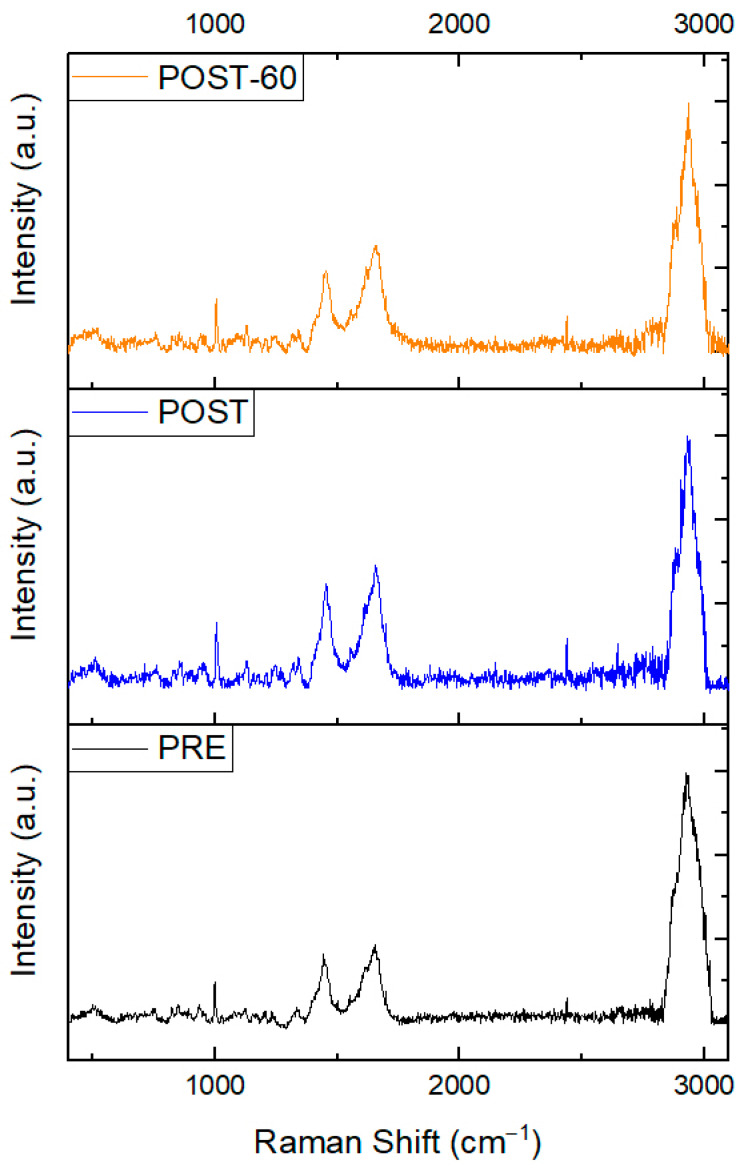
Mean normalized Raman spectra collected before shearing (PRE), immediately after the shearing procedure (POST), and after 60 min at the end of the shearing practice (POST60).

Figure 1 presents the mean spectrum of sheep serum from samples used in this study and collected before shearing (PRE), immediately after the shearing procedure (POST), and after 60 min at the end of the shearing (POST60). All spectra exhibit the main typical vibrational modes of proteins.

**Figure 2:**
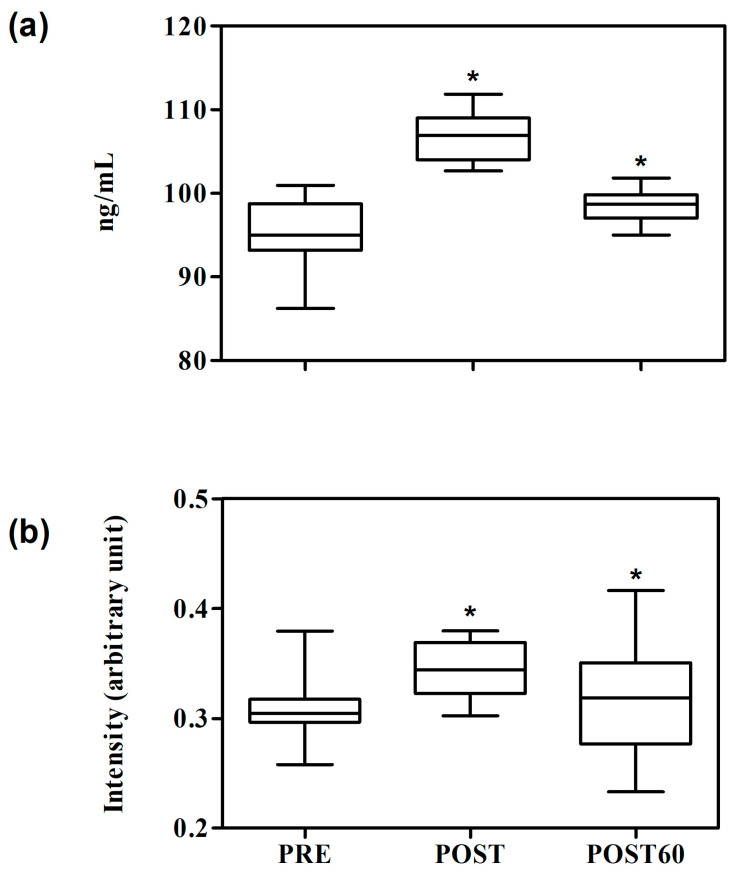
Boxplot of cortisol serum levels recorded using an ELISA test (**a**) and Raman cortisol areas of the 1300–1366 cm^−1^ band (**b**) obtained from sera before shearing (PRE), immediately after the shearing (POST), and after 60 min after the end of the shearing (POST60). The median value is highlighted by a horizontal line, and the mean value is depicted as a square. * indicates statistical differences versus the previous data points.

The application of one-way repeated measures ANOVA to cortisol serum values obtained using ELISA analysis and Raman spectroscopy revealed statistically significant differences among the data points (*p* < 0.0001). In particular, all data points were statistically different from the others; the highest value was observed after the shearing, as shown in Figure 2.

Figure 2 shows the boxplot analysis of the temporal cortisol variation profile evaluated by the ELISA test (Figure 2a) and the Raman spectroscopy technique (Figure 2b). All Raman intensity values are indicated in arbitrary units (A.U.). Group differences were first tested using a one-way ANOVA, followed by Bonferroni post-hoc comparisons in order to assess pairwise differences (PRE vs. POST, PRE vs. POST60, POST vs. POST60). At baseline (PRE), the ELISA median cortisol value was 94.98 ng/mL, with relatively narrow dispersion across individuals. The corresponding Raman intensities displayed median values of 0.308 A.U., with moderate variability. Five minutes after post-shearing (POST), both methods revealed a marked increase. ELISA cortisol concentrations rose significantly, reaching a median of 104 ng/mL (*p* < 0.001), with relatively tight clustering of the data, indicating a consistent endocrine response across the sampled animals. In parallel, Raman intensities increased to a median value of 0.35 A.U., also significantly higher compared to baseline (*p* < 0.05). The magnitude of change was similar in direction to that observed with ELISA, although the spread of the Raman values was slightly broader.

At 60 min post-shearing (POST60), both assays showed a decline compared to the immediate post-shearing peak. ELISA cortisol values decreased towards 98.70 ng/mL, remaining elevated compared to baseline (*p* < 0.01) but clearly lower than POST (*p* < 0.001). Raman intensities followed the same downward trajectory, returning to 0.32 A.U., which was not different from PRE (*p* > 0.05) but lower than POST (*p* < 0.5). Interestingly, Raman boxplots revealed a wider interquartile range at this later time point, suggesting that while the overall trend mirrored the ELISA, individual responses captured by Raman spectra were more heterogeneous. However, taken together, the boxplot distributions highlight a strong agreement between the two approaches, with both detecting the acute cortisol surge following shearing and its subsequent partial recovery within 60 min.

**Figure 3:**
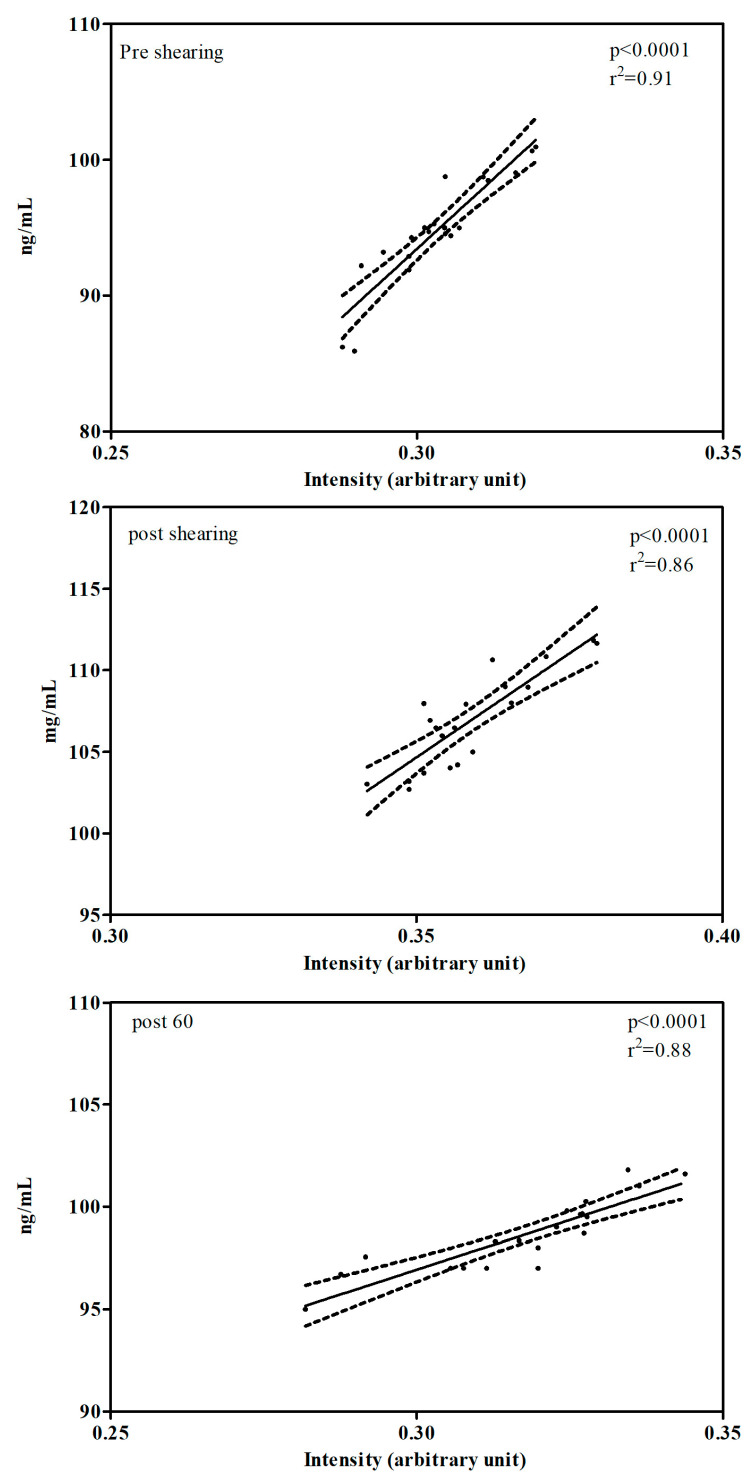
Correlation between the cortisol values obtained by the ELISA and Raman spectroscopy assessments observed during the various data points, before shearing (PRE), immediately after the shearing (POST), and after 60 min after the end of the shearing (POST60). Solid lines represent the linear regression (the regression coefficients r^2^ is reported in the upper right corner of each figure), whereas dot lines indicate the 95% confidence interval.

A positive correlation between the cortisol values obtained by ELISA and Raman spectroscopy assessments at all data points (PRE *p* < 0.0001, r^2^ = 0.91; POST *p* < 0.0001, r^2^ = 0.86; POST60 *p* < 0.0001, r^2^ = 0.88) was observed (Figure 3).

## 4. Discussion

The findings of the present study clearly show that, compared to pre-shearing values, the fleece removal procedure induces a significant increase in serum cortisol levels. This evidence is consistent with previous studies, some of which were conducted on plasma samples [48,49,50,51,52,53,54], confirming that shearing represents a significant source of stress for the animal.

Baseline serum cortisol concentrations in sheep, before shearing procedures, show considerable variability across studies, generally ranging from approximately 8 to 95 ng/mL and even more [49,54,55]. Such differences are influenced by multiple factors, including breed, reproductive status, environmental conditions, handling, and even the time of day at which blood sampling is performed, due to the circadian rhythm of cortisol secretion. Specifically, our study revealed a marked increase in serum cortisol concentrations 5 min after shearing (POST), followed by a partial reduction at 60 min (POST60). Despite this decrease, the levels remained higher than those measured during the pre-shearing period (PRE).

An interesting finding of this study concerns the trend in serum cortisol levels at 60 min post-shearing (POST60), which were lower than those recorded at 5 min (POST). The temporal profile observed, with a sharp peak at 5 min and a partial return to baseline after 60 min, reflects the physiological dynamics of cortisol as part of the acute stress response. Such responses are central to the concept of allostasis, whereby animals adapt to stressors to maintain homeostasis. The perception of and response to stress can vary from one subject to another depending on genetic background, past experiences, physiological context, and the timing with which the organism adapts to the stimulus and re-establishes homeostasis. From a welfare perspective, monitoring these hormonal responses provides objective indicators of animal well-being, which are crucial for the development of ethical and sustainable farming practices [56,57,58].

Although blood sampling itself can represent a potentially stressful stimulus, capable of activating the hypothalamic–pituitary–adrenal (HPA) axis and triggering a transient secretion of glucocorticoids, particularly cortisol [55], the present study clearly demonstrated that shearing induces a more pronounced endocrine response. Serum cortisol levels increased significantly after the shearing, indicating that the procedure constitutes a substantial stressor for the animal. It is plausible that the acute stress response observed was not attributable to a single factor, but rather to the combination of multiple elements intrinsic to the shearing procedure, including fear caused by human approach (perceived as a threat), capture, restraint, isolation from the flock, immobilization, and direct physical handling, all of which may contribute to elevated serum cortisol concentrations [49]. Building on these physiological considerations, the present study was designed with the main objective of evaluating the effectiveness of Raman spectroscopy in detecting such changes in serum cortisol concentrations in sheep subjected to shearing. Alongside the results obtained through one-way repeated measurements ANOVA on the ELISA data, an ANOVA applied to the spectral areas of the cortisol band obtained through Raman spectroscopy also showed statistically significant differences among the three experimental conditions (PRE, POST, POST60), displaying an overall trend that was consistent with that observed using the ELISA method.

A particularly noteworthy aspect of this study is the strong positive correlation found between cortisol values obtained via an ELISA and those derived from Raman spectroscopy at all three time points. This result highlights the reliability and validity of Raman spectroscopy in identifying changes in cortisol levels, supporting its use as an alternative or complementary method for stress monitoring.

The methodological comparison between ELISA and Raman deserves attention. An ELISA remains the gold standard for hormone quantification, offering validated kits with high sensitivity and specificity, but requiring several hours, skilled operators, and consumables. Raman spectroscopy, in contrast, is a fast, non-destructive technique that enables rapid measurements without complex sample preparation, relying only on optical interrogation of microliter volumes, and it does not require chemical reagents. Compared to traditional immunoenzymatic tests, which require costly commercial kits, it is considerably more economical and advantageous.

Recent studies have documented the possibility of identifying and quantifying endogenous molecules such as cortisol in human and ovine serum [59,60]. Our work helps to strengthen this evidence, demonstrating strong agreement between the Raman spectroscopy signal and the serum levels measured through immunoenzymatic assays.

Most Raman-based studies on cortisol have relied on SERS (Surface-Enhanced Raman Spectroscopy), which employs plasmonic substrates to amplify weak Raman signals and allows the detection of target molecules at very low concentrations [40,59,61].

SERS spectra can sometimes differ from normal Raman spectra, potentially showing broader bands or peaks attributed to the substrate itself, which requires users to create their own SERS spectral databases for accurate analysis. In fact, SERS enhances different vibrational modes of a molecule to varying degrees, depending on the orientation of the molecule relative to the SERS substrate. This is known as the surface selection rule. For example, a vibrational mode perpendicular to the metal surface might be enhanced more than the parallel one. As a result, the relative intensities of the peaks in a SERS spectrum are often different from the relative intensities in a normal Raman spectrum. However, the uncertainty levels are in terms of peak intensity rather than in the peak position. For this reason, we consider the peak centered at around 1330 cm^−1^ reliable for cortisol evaluation and focus our study on the 1300–1366 cm^−1^ band analysis [40,41]. While highly sensitive, SERS protocols often suffer from reproducibility issues, and the production and maintenance of reliable SERS substrates can be costly and technically demanding, which represents a further limitation for their large-scale or routine application [62,63]. In contrast, our study employed conventional Raman spectroscopy, without metallic enhancement, demonstrating that cortisol-associated spectral variations could be detected directly in ovine serum. Although this approach is inherently less specific and sensitive than SERS, its simplicity, reproducibility, and label-free nature make it attractive for translational and veterinary applications.

Several limitations of our study should be acknowledged. First, the sample size was relatively modest, which may limit the generalizability and the robustness of our results. Although the animals were homogeneous in terms of age and sex, the restricted number of subjects may still limit the representativeness of the findings. In a small cohort, even slight individual variations in endocrine activity or responsiveness to handling can exert an important influence on the results, making the overall interpretation more vulnerable to variability. Finally, as previously described, conventional Raman spectroscopy inherently has lower sensitivity compared to SERS, limiting its ability to detect very low hormone concentrations.

Despite these limitations, our study presents significant strengths. It demonstrates that label-free, conventional Raman spectroscopy, without the need for plasmonic substrates, can reliably detect cortisol-related spectral changes directly in ovine serum. The method’s simplicity, cost-effectiveness, minimal sample handling, and higher reproducibility support its use as a practical complementary tool to immunoassay-based techniques.

Future research should extend the application of Raman spectroscopy to alternative biological matrices, such as saliva, milk, or wool, which would enable non-invasive stress monitoring. Combining Raman with behavioral and physiological indicators (e.g., heart rate variability, infrared thermography) could provide a holistic assessment of stress. Moreover, it would be valuable to replicate the present study using a larger cohort of sheep to improve statistical power and SERS-based methodologies, which, despite their current limitations in reproducibility, could potentially enhance sensitivity and allow detection of cortisol at lower concentrations. Finally, another line of investigation could be to extend the analysis to additional biomarkers of stress, such as adrenocorticotropic hormone (ACTH), catecholamines, cortisone, or acute-phase proteins, thereby providing a more comprehensive evaluation of the HPA axis and related physiological pathways.

## 5. Conclusions

This feasibility study aimed to evaluate the ability of conventional, label-free Raman spectroscopy to detect variations in serum cortisol concentrations in sheep subjected to shearing, and to compare these results with those obtained through ELISA, the current gold standard. Our findings demonstrated that Raman analysis detected significant temporal changes, with cortisol levels rising immediately after shearing (POST) and partially declining after 60 min (POST60), in line with the dynamics observed by ELISA. This consistency supports the potential of Raman spectroscopy as a complementary tool for stress monitoring in sheep, offering advantages such as rapid analysis, minimal sample preparation, and reproducibility without the need for costly reagents. While this preliminary feasibility study was limited by the relatively small sample size, the focus on a single stressor, and the analysis of only one biomarker, the results provide proof-of-concept evidence for the application of Raman spectroscopy in veterinary endocrinology. Further studies should validate these findings in larger cohorts, explore other stress-related biomarkers, and assess alternative biological matrices, thereby expanding the utility of this technique for animal welfare assessment.

## Figures and Tables

**Table 1:** Raman center frequency, tentative assignment and related reference of the main vibrational bands and peaks of serum in the 400–3020 cm^−1^ range.

Center Frequency (cm^−1^)	Tentative Assignment	References
520	Disulfide band	[42]
759	Ring vibration of tryptophan	[36,43]
830 and 850	Tyrosine doublet	[37]
1000	Phenylalanine	[42,44]
1213–1279 band	Leucine and isoleucine	[43,45]
1300–1366 band	C-C-C symmetric stretching in the Cortisol A-ring	[40,41]
1450 band	CH2 scissoring deformation	[46]
1550	Amide II vibration	[42]
1650	Amide I vibration	[34]
2930	C-H stretching vibration	[34,47]

## Data Availability

The original data presented in the study are openly available in MendeleyData at DOI: 10.17632/s37fyfyhpp.1.
